# Supplementary material for: Intricate microbiome differences observed in lactating cows across methane intensity phenotypes
Source: ISME Commun. 2026 Jun 7;6(1):ycag155. doi: 10.1093/ismeco/ycag155 (PMC13431278; doi:10.1093/ismeco/ycag155)
Supplement: Supplementary_material_ycag155 [file supplementary_material_ycag155.zip › SF_12.pdf]

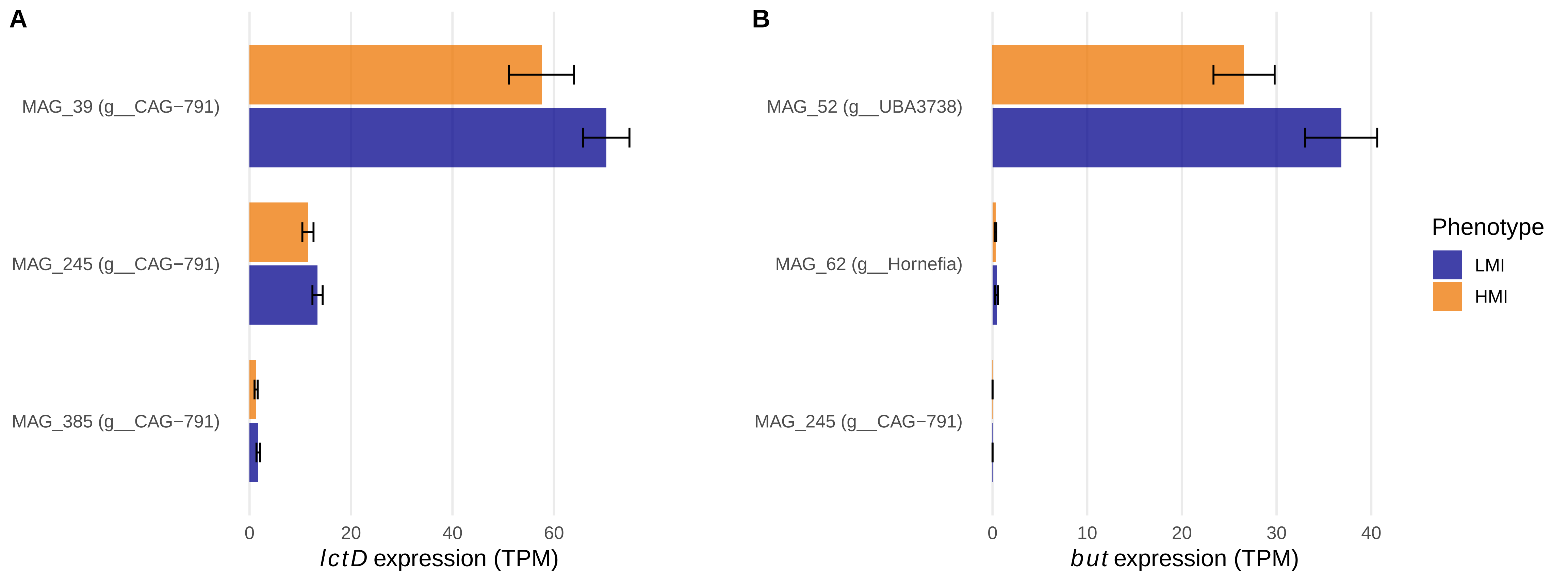

A
MAG_39 (g__CAG−791)
MAG_245 (g__CAG−791)
MAG_385 (g__CAG−791)
0
20
40
60
lctD expression (TPM)
B
MAG_52 (g__UBA3738)
MAG_62 (g__Hornefia)
MAG_245 (g__CAG−791)
0
10
20
30
40
but expression (TPM)
Phenotype
LMI
HMI
