# Supplementary material for: Intricate microbiome differences observed in lactating cows across methane intensity phenotypes
Source: ISME Commun. 2026 Jun 7;6(1):ycag155. doi: 10.1093/ismeco/ycag155 (PMC13431278; doi:10.1093/ismeco/ycag155)
Supplement: Supplementary_material_ycag155 [file supplementary_material_ycag155.zip › SF_5.pdf]

Substrate	CAZy	MAG_7 LMI	MAG_7 HMI	MAG_277 LMI	MAG_277 HMI	MAG_153 LMI	MAG_153 HMI	MAG_145 LMI	MAG_145 HMI	MAG_346 LMI	MAG_346 HMI	MAG_193 LMI	MAG_193 HMI	MAG_228 LMI	MAG_228 HMI	MAG_368 LMI	MAG_368 HMI	MAG_150 LMI	MAG_150 HMI
Xylan	CE1			1.62	1.61					0.07	0.06	0.70	0.61					0.10	0.12
Xylan	CE2	0.38	0.44	2.20	1.68			2.14	2.11	0.66	0.78			1.29	2.36				
Xylan	GH10			5.46	4.30			3.41	4.00	0.58	0.69	0.28	0.17	5.40	6.07			1.05	1.34
Xylan	GH11																	0.73	0.77
Xylan	GH115	0.35	0.41	0.79	0.63			0.42	0.50	0.20	0.26			0.32	0.48				
Xylan	GH43	0.27	0.21	2.08	2.08	1.72	2.37	1.32	1.61	0.78	1.05	0.55	0.45	1.31	1.71	0.10	0.08	0.17	0.18
Xylan	GH67					0.82	0.93							1.45	2.35				
Xylan	GH8					0.31	0.80			0.30	0.33			0.59	0.94			0.93	0.93
Cellulose	GH3	0.11	0.11	2.02	1.74	0.91	0.91	0.61	0.89	0.70	0.83	0.67	0.52	2.00	3.10				
Cellulose	GH45																	1.63	1.75
Cellulose	GH5	0.15	0.19	1.54	1.41	0.37	0.37	1.67	2.55	0.62	0.60	3.30	2.49	0.96	1.42			0.87	1.01
Cellulose	GH9	0.36	0.51	2.57	2.62									1.74	2.07			0.31	0.33
Hemicellulose	GH105	0.48	0.49	1.68	1.72					0.17	0.32			0.22	0.25	0.42	0.37		
Hemicellulose	GH106	0.83	0.95	1.30	1.02					0.10	0.10								
Hemicellulose	GH16			0.29	0.20					0.15	0.17	0.91	1.04					5.79	5.22
Hemicellulose	GH2	0.38	0.43	1.72	1.58	0.41	0.40	0.32	0.46	0.40	0.50	0.34	0.32	0.77	1.11	0.40	0.34		
Hemicellulose	GH26	0.18	0.11	0.95	0.79	0.45	0.48	0.10	0.04					1.01	1.43			0.46	0.63
Hemicellulose	GH27																	0.50	0.42
Hemicellulose	GH35	0.43	0.31	1.88	1.79	1.19	1.06			0.50	0.48			1.00	1.54				
Hemicellulose	GH36	0.42	0.42	3.78	3.02			1.57	2.46	0.66	0.64	1.21	0.93	2.38	3.12				
Hemicellulose	GH38									0.27	0.29								
Hemicellulose	GH78			0.47	0.75									1.41	1.95				
Hemicellulose	GH97	0.69	0.59	0.83	0.91	0.64	0.67	0.69	0.90	0.77	0.79	0.83	0.48	2.18	3.09				
Hemicellulose	PL11	0.14	0.10							0.02	0.03	0.96	0.75	0.87	1.11			1.26	1.33
Pectin	CE12	0.21	0.31	1.81	2.01					0.21	0.27	0.53	0.31	0.53	1.22			0.32	0.21
Pectin	CE8	0.36	0.40	0.95	1.04			0.92	1.05					0.83	1.79			0.08	0.15
Pectin	GH28	1.20	1.35	1.96	1.72	1.74	2.16	0.49	0.50	1.30	1.83	0.21	0.09	2.16	3.84				
Pectin	GH95	0.41	0.41	1.16	1.36	0.54	0.83	0.68	0.77	0.62	0.60	0.64	0.50	1.20	1.62				
Pectin	PL1	1.04	1.23			3.04	2.76			0.28	0.16	0.44	0.33	0.98	1.96			0.29	0.35
Pectin	PL10																	0.14	0.22
Starch	GH13	0.89	0.93	4.29	3.93	0.97	0.93	2.16	2.38	1.53	1.63			3.20	4.31	0.33	0.18	0.28	0.34
Starch	GH31	0.07	0.09	3.85	3.49	0.84	0.97	0.25	0.24	0.33	0.30	0.72	0.59	1.73	2.52				
Starch	GH57	5.97	5.08	23.23	18.89	10.51	11.34					4.43	2.42	2.66	3.16			0.06	0.11
Chitin	GH18																	0.46	0.56
Chitin	GH20			0.81	0.70	0.66	1.03												
Arabinan	GH127	1.77	1.64									0.45	0.31						
Arabinan	GH30	0.15	0.12				0.05					0.70	0.50					0.14	0.11
Arabinan	GH39																	0.31	0.76
Arabinan	GH51	0.20	0.23	5.31	4.65	4.32	4.39	1.27	1.76	1.40	1.52	1.23	1.26	3.61	4.43				0.01
Arabinan	GH53	0.23	0.28	2.16	2.16			1.65	3.18	0.41	0.81			2.41	3.33			0.15	0.15
Fructan	GH32			4.29	2.95			1.37	2.07	1.12	0.89	0.43	0.32						

Substrate	CAZy	MAG_381 LMI	MAG_381 HMI	MAG_335 LMI	MAG_335 HMI	MAG_39 LMI	MAG_39 HMI	MAG_104 LMI	MAG_104 HMI	MAG_312 LMI	MAG_312 HMI	MAG_125 LMI	MAG_125 HMI	MAG_232 LMI	MAG_232 HMI	MAG_49 LMI	MAG_49 HMI	MAG_167 LMI	MAG_167 HMI
Xylan	CE1	0.09	0.04							0.82	0.95	0.05	0.03	0.60	0.74				
Xylan	CE2									0.58	1.16		0.14					1.24	1.29
Xylan	GH10	2.15	3.15							2.16	2.16	0.21	0.16	1.96	1.69			1.74	1.23
Xylan	GH11																		
Xylan	GH115			0.95	0.95					0.37	0.42	0.20	0.15	0.63	0.88			0.40	0.52
Xylan	GH43	0.11	0.12	0.48	0.49			2.94	3.07	2.16	1.71	0.13	0.10	0.85	0.86	0.17	0.17	1.13	1.01
Xylan	GH67			0.65	0.83							0.12	0.17					1.17	0.98
Xylan	GH8	1.09	1.73																
Cellulose	GH3	0.65	1.41	0.49	0.83	1.01	0.86	3.49	3.55	0.77	0.73	0.17	0.16	0.64	0.60	0.70	0.76	0.65	0.66
Cellulose	GH45	2.08	2.50																
Cellulose	GH5	0.93	1.48	0.22	0.34			1.99	1.70	2.65	2.62	0.33	0.25	1.94	1.82			0.68	0.65
Cellulose	GH9	0.24	0.37							0.31	0.28								
Hemicellulose	GH105			0.52	0.59													0.48	0.55
Hemicellulose	GH106			0.86	1.16													0.35	0.41
Hemicellulose	GH16	6.18	9.75							0.22	0.15			0.04	0.10			0.04	0.01
Hemicellulose	GH2	0.34	0.58	0.50	0.73	0.97	0.76	2.36	2.13	0.65	0.65	0.16	0.15	0.48	0.57	0.07	0.10	0.75	0.66
Hemicellulose	GH26	0.80	1.05							1.06	0.96			0.32	0.57			0.88	0.60
Hemicellulose	GH27													0.25	0.37				
Hemicellulose	GH35			0.44	0.36									0.90	0.74				
Hemicellulose	GH36			0.74	1.06	0.83	1.14	3.74	3.84	1.22	1.07					0.74	0.46	1.41	1.12
Hemicellulose	GH38																		
Hemicellulose	GH78			0.61	0.49			0.08	0.09									0.76	0.58
Hemicellulose	GH97			0.49	0.63					1.13	1.36	0.18	0.15	1.66	1.56			0.62	0.56
Hemicellulose	PL11			0.66	0.90														
Pectin	CE12			0.60	0.51									0.97	1.23			0.60	0.59
Pectin	CE8			0.15	0.26					0.11	0.27			0.97	0.82			0.68	0.48
Pectin	GH28			1.28	1.53							0.15	0.11	0.49	0.50			1.42	1.28
Pectin	GH95	0.09	0.42							0.71	0.72	0.09	0.07	0.61	0.54			1.06	0.83
Pectin	PL1			0.05	0.04									1.97	1.37			0.64	0.74
Pectin	PL10			0.52	0.87													0.27	0.24
Starch	GH13	0.29	0.33	3.83	5.00	2.79	2.36	2.83	3.02	2.10	2.24	0.17	0.14	1.59	1.86	0.19	0.10	2.19	1.85
Starch	GH31			0.36	0.54			1.84	1.65	0.57	0.78	0.08	0.08	0.84	0.76			1.08	0.82
Starch	GH57	0.49	0.51									0.58	0.59	5.22	5.07			10.00	8.82
Chitin	GH18	1.79	2.80			1.16	0.96					0.19	0.27						
Chitin	GH20											0.18	0.09						
Arabinan	GH127			0.84	1.39							0.05	0.03					0.93	0.97
Arabinan	GH30	0.21	0.28							1.95	1.82			1.18	1.61				
Arabinan	GH39																		
Arabinan	GH51	0.14	0.22					9.14	8.49	4.13	3.32	0.11	0.10	1.76	2.03			1.02	0.92
Arabinan	GH53									0.64	0.91			0.33	0.28			0.73	0.70
Fructan	GH32			1.72	1.47	1.48	1.07					1.21	0.92	2.30	1.47			0.52	0.36

Scaled fold−change (log10): 1.0, 0.5
