# Supplementary material for: Intricate microbiome differences observed in lactating cows across methane intensity phenotypes
Source: ISME Commun. 2026 Jun 7;6(1):ycag155. doi: 10.1093/ismeco/ycag155 (PMC13431278; doi:10.1093/ismeco/ycag155)

		MAG_39		MAG_21		MAG_113		MAG_104		MAG_407		MAG_25		MAG_15		MAG_314		MAG_68		MAG_140		MAG_331		MAG_293		MAG_58		MAG_189		MAG_228		MAG_277		MAG_7	
		LMI	HMI	LMI	HMI	LMI	HMI	LMI	HMI	LMI	HMI	LMI	HMI	LMI	HMI	LMI	HMI	LMI	HMI	LMI	HMI	LMI	HMI	LMI	HMI	LMI	HMI	LMI	HMI	LMI	HMI	LMI	HMI	LMI	HMI
Xylan	CE1																					5.07	5.47									1.62	1.61		
	CE2																													1.29	2.36	2.20	1.68	0.38	0.44
	GH10																					14.00	15.07							5.40	6.07	5.46	4.30		
	GH115																					0.71	0.88							0.32	0.48	0.79	0.63	0.35	0.41
	GH43					0.22	0.26	2.94	3.07									3.86	1.30			2.61	2.76	1.52	2.25					1.31	1.71	2.08	2.08	0.27	0.21
	GH67																					6.57	8.03							1.45	2.35				
	GH8																					1.51	1.53							0.59	0.94				
Cellulose	GH3	1.01	0.86	7.08	8.22	2.99	3.51	3.49	3.55	0.36	0.35	1.49	1.81	0.25	0.44	0.43	0.30	2.46	1.00	3.50	2.89	1.37	1.70	2.86	4.88	1.07	0.86	2.17	2.37	2.00	3.10	2.02	1.74	0.11	0.11
	GH5					0.09	0.09	1.99	1.70	1.06	1.41			1.00	1.35			1.98	1.07							0.39	0.66			0.96	1.42	1.54	1.41	0.15	0.19
	GH9																													1.74	2.07	2.57	2.62	0.36	0.51
Hemicellulose	GH105			4.03	6.28	2.38	2.94													7.49	4.93	2.11	2.12					10.63	14.36	0.22	0.25	1.68	1.72	0.48	0.49
	GH106													0.05	0.23	0.21	0.18					0.74	0.61									1.30	1.02	0.83	0.95
	GH16																															0.29	0.20		
	GH2	0.97	0.76	2.50	3.04	1.97	2.22	2.36	2.13	1.15	1.26	0.45	0.63	0.54	0.58	2.01	1.32	1.71	0.80	0.93	0.81	1.25	1.42	0.42	1.02	0.82	0.65			0.77	1.11	1.72	1.58	0.38	0.43
	GH26																													1.01	1.43	0.95	0.79	0.18	0.11
	GH27					0.35	0.16					0.23	0.21									1.87	1.84			0.16	0.11								
	GH35					3.04	4.78							0.06								1.89	2.12							1.00	1.54	1.88	1.79	0.43	0.31
	GH36	0.83	1.14	2.63	3.36	3.39	5.17	3.74	3.84	0.38	0.31	1.84	3.77			2.04	1.23			3.67	3.58			2.65	4.41	2.24	3.40			2.38	3.12	3.78	3.02	0.42	0.42
	GH78					1.66	2.32	0.08	0.09			0.04	0.02	0.08	0.17					1.58	1.29	0.75	0.41	0.57	0.92					1.41	1.95	0.47	0.75		
	GH97																					2.10	2.34							2.18	3.09	0.83	0.91	0.69	0.59
	PL11																					1.19	1.32							0.87	1.11			0.14	0.10
Pectin	CE12																					0.98	0.75							0.53	1.22	1.81	2.01	0.21	0.31
	CE8																					2.12	2.59							0.83	1.79	0.95	1.04	0.36	0.40
	GH28			5.82	7.74	3.15	3.67													3.45	2.37	1.83	2.25					8.65	9.90	2.16	3.84	1.96	1.72	1.20	1.35
	GH42					0.04	0.07																												
	GH95																													1.20	1.62	1.16	1.36	0.41	0.41
	PL1																					1.35	1.32							0.98	1.96			1.04	1.23
	PL9			4.00	4.56																														
Starch	GH13	2.79	2.36	3.80	4.50	2.03	2.31	2.83	3.02	12.88	11.39	2.68	4.21	4.25	5.29	1.54	1.23	1.97	0.88	4.57	3.69	3.00	3.47	3.83	5.57	2.03	1.73	2.17	2.65	3.20	4.31	4.29	3.93	0.89	0.93
	GH31							1.84	1.65							1.08	0.82	0.93	0.53			2.71	3.11			1.09	0.68	1.04	0.99	1.73	2.52	3.85	3.49	0.07	0.09
	GH57																					27.53	28.45							2.66	3.16	23.23	18.89	5.97	5.08
Chitin	GH18	1.16	0.96	3.02	3.58	5.63	5.90					1.12	1.81							1.55	1.47			1.36	2.66	1.68	0.80								
	GH20																															0.81	0.70		
Arabinan	GH127																					2.05	2.06											1.77	1.64
	GH30																					0.79	1.24											0.15	0.12
	GH51					0.84	0.98	9.14	8.49													1.60	1.89							3.61	4.43	5.31	4.65	0.20	0.23
	GH53																			0.96	0.81	0.71	0.66							2.41	3.33	2.16	2.16	0.23	0.28
Fructan	GH32	1.48	1.07	24.76	30.47	7.25	7.94			13.62	12.10	2.51	4.18	6.98	9.37	11.29	8.63	8.88	3.60	9.48	8.45	1.29	1.37	4.51	7.97	1.80	1.70					4.29	2.95		

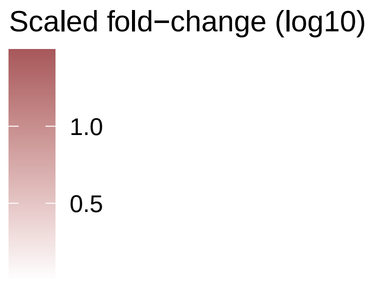

Supplement: Supplementary_material_ycag155 [file supplementary_material_ycag155.zip › SF_6.pdf]
