# Supplementary material for: Intricate microbiome differences observed in lactating cows across methane intensity phenotypes
Source: ISME Commun. 2026 Jun 7;6(1):ycag155. doi: 10.1093/ismeco/ycag155 (PMC13431278; doi:10.1093/ismeco/ycag155)
Supplement: Supplementary_material_ycag155 [file supplementary_material_ycag155.zip › SF_8.pdf]

KO	Pathway	MAG_39		MAG_21		MAG_113		MAG_104		MAG_407		MAG_174		MAG_25		MAG_15		MAG_314		MAG_68	
		LMI	HMI	LMI	HMI	LMI	HMI	LMI	HMI	LMI	HMI	LMI	HMI	LMI	HMI	LMI	HMI	LMI	HMI	LMI	HMI
K00169	Acetate	1.65	1.19									2.36	3.19								
K00170		1.19	0.87									1.16	2.71								
K00171		0.71	0.59									1.03	1.25								
K00172		1.05	1.11									1.74	3.51								
K00625				45.97	51.98	40.74	48.99			13.05	14.83							6.30	4.59	6.09	3.24
K00656				18.52	23.09																
K00925		113.16	89.27	33.03	39.04	42.86	51.16							12.69	20.39	6.68	9.92				
K03737		284.79	223.39	76.41	86.36	116.13	128.12	34.19	30.71	94.77	86.17			30.64	45.14	22.49	29.10	27.66	19.55	23.48	9.61
K00016	Lactate					2.71	3.30							15.92	28.58						
K03778				1.15	0.83	40.50	41.98							32.63	54.51					127.26	59.12
K19266												0.14	0.22								
K17992	WLP_additional			43.12	55.11											6.23	7.82				
K18331				35.87	40.75																
K18332				49.44	54.88	16.87	19.66							4.56	6.93	8.43	11.98				
K00016	Propionate via acrylate					2.71	3.30							15.92	28.58						
K01026				1.80	1.76									3.56	4.86						
K00123	WLP	3.02	2.37																		
K00125												10.96	14.08								
K00297				2.37	1.43	1.76	2.04			1.06	0.75			1.28	1.32	0.35	0.32	1.41	1.19	2.51	1.07
K01491																					
K01500				0.92	1.02	1.30	2.39			0.88	0.79					0.27	0.49			1.14	0.45
K01938		5.74	5.40	9.86	10.24	1.88	2.61	5.55	5.04	2.01	1.75			20.62	30.38	0.91	1.12			1.26	0.65
K22516												39.68	52.34								
K00248	Butyrate	92.94	77.83			56.38	63.64							51.36	80.83						
K00626		211.57	172.62			44.16	48.02							49.61	81.74						
K00929										7.94	7.08					1.02	1.06				
K01715		1.80	1.30			53.99	63.05							32.12	54.95						
K04072	Ethanol			21.03	27.23			3.25	2.99	26.60	30.34							56.27	46.04		
K00239	Propionate via succinate																				
K00240										0.09			0.05		0.08	0.65	0.55			0.33	0.12
K00241																					
K01676																					
K01677				1.37	1.41							0.09	0.05								
K01678					0.04																
K01679		2.68	2.09			3.12	3.72	1.53	1.02					1.53	2.79	0.57	1.16	0.42	0.51	0.88	0.38
K01847																					
K01902																					
K01958		40.70	31.56																		

KO	Pathway	MAG_140		MAG_331		MAG_293		MAG_58		MAG_189		MAG_228		MAG_277		MAG_83		MAG_10		MAG_7	
		LMI	HMI	LMI	HMI	LMI	HMI	LMI	HMI	LMI	HMI	LMI	HMI	LMI	HMI	LMI	HMI	LMI	HMI	LMI	HMI
K00169	Acetate							0.21	0.16							0.85	1.23	1.97	2.34		
K00170								0.17	0.15							1.12	1.08	2.77	2.98		
K00171								0.05	0.27							0.43	0.41	1.50	1.45		
K00172								0.51	0.79							0.37	0.95	0.48	1.22		
K00625		13.05	10.40			0.72	0.93														
K00656						0.06	0.22													3.33	3.09
K00925		28.38	21.91	11.01	12.36			15.43	13.76	11.87	12.68	7.22	8.23							5.77	5.12
K03737		12.12	9.95	22.02	25.35	33.39	40.33	113.33	90.83			26.24	32.92	34.81	28.65					25.90	26.51
K00016	Lactate							14.62	12.38												
K03778				3.01	4.60			27.27	23.24												
K19266																		0.09	0.25		
K17992	WLP_additional																				
K18331																					
K18332								7.79	6.93												
K00016	Propionate via acrylate							14.62	12.38												
K01026										1.15	1.36										
K00123	WLP																				
K00125																		23.41	24.06		
K00297		2.17	1.39	1.47	1.25			0.82	1.08			2.86	3.58							2.10	2.35
K01491								1.11	1.00			2.66	3.40	2.43	2.02					2.67	2.73
K01500		6.91	5.86			2.04	2.82														
K01938		7.88	5.92					0.84	0.85												
K22516																		40.38	45.83		
K00248	Butyrate							77.04	65.76												
K00626								25.01	20.60												
K00929		1.72	1.06	1.77	2.07	0.18	0.52							2.22	1.66						
K01715								47.51	42.33												
K04072	Ethanol	63.60	62.87																		
K00239	Propionate via succinate			26.61	28.88									38.37	33.31					21.86	16.75
K00240				26.57	28.35									41.22	27.89			0.14	0.11	16.43	12.44
K00241				27.15	30.17									44.10	38.23					21.03	19.07
K01676				7.18	8.44							9.88	10.80							4.02	3.76
K01677																0.14	0.14	0.33	0.25		
K01678																0.22	0.32	0.22	0.05		
K01679		1.38	0.92					2.23	2.23	0.97	1.53										
K01847												11.67	15.41	13.48	11.89						
K01902																0.63	0.73	0.60	0.44		
K01958																					

Scaled fold−change (log10)
2.0
1.5
1.0
0.5
