# Supplementary material for: Intricate microbiome differences observed in lactating cows across methane intensity phenotypes
Source: ISME Commun. 2026 Jun 7;6(1):ycag155. doi: 10.1093/ismeco/ycag155 (PMC13431278; doi:10.1093/ismeco/ycag155)
Supplement: Supplementary_material_ycag155 [file supplementary_material_ycag155.zip › SF_9.pdf]

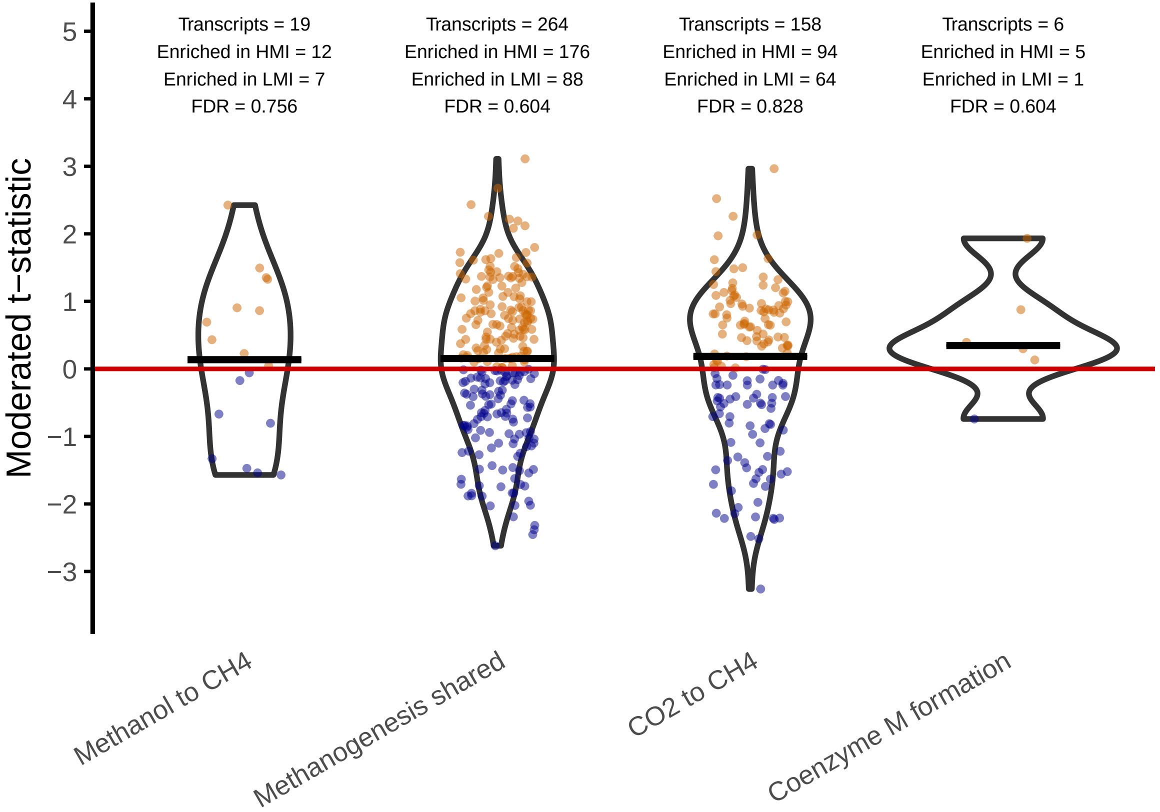

Transcripts = 19
Enriched in HMI = 12
Enriched in LMI = 7
FDR = 0.756
Transcripts = 264
Enriched in HMI = 176
Enriched in LMI = 88
FDR = 0.604
Transcripts = 158
Enriched in HMI = 94
Enriched in LMI = 64
FDR = 0.828
Transcripts = 6
Enriched in HMI = 5
Enriched in LMI = 1
FDR = 0.604
Moderated t−statistic
5
4
3
2
1
0
−1
−2
−3
Methanol to CH4
Methanogenesis shared
CO2 to CH4
Coenzyme M formation
