# Supplementary material for: Intricate microbiome differences observed in lactating cows across methane intensity phenotypes
Source: ISME Commun. 2026 Jun 7;6(1):ycag155. doi: 10.1093/ismeco/ycag155 (PMC13431278; doi:10.1093/ismeco/ycag155)
Supplement: Supplementary_material_ycag155 [file supplementary_material_ycag155.zip › SF_10.pdf]

Family	Substrate	MAG_385		MAG_52		MAG_245		MAG_39		MAG_62		MAG_44		MAG_68		MAG_146		MAG_314		MAG_195		MAG_228		MAG_217	
		LMI	HMI	LMI	HMI	LMI	HMI	LMI	HMI	LMI	HMI	LMI	HMI	LMI	HMI	LMI	HMI	LMI	HMI	LMI	HMI	LMI	HMI	LMI	HMI
CE1	Xylan															0.34	0.13								
CE2	Xylan																					1.29	2.36	0.08	2.31
GH10	Xylan															3.82	2.18			0.37	0.47	5.40	6.07	0.19	3.06
GH11	Xylan															0.49	0.20								
GH115	Xylan																			0.22	0.30	0.32	0.48	0.06	2.27
GH43	Xylan													3.86	1.30	0.24	0.15			0.19	0.30	1.31	1.71	0.12	2.63
GH67	Xylan																			0.66	0.73	1.45	2.35	0.04	1.47
GH8	Xylan																					0.59	0.94		
GH3	Cellulose	0.80	0.69					1.01	0.86	0.23	0.19			2.46	1.00	0.76	0.38	0.43	0.30	1.04	1.07	2.00	3.10	0.19	2.04
GH48	Cellulose															27.67	16.92								
GH5	Cellulose											1.35	0.83	1.98	1.07	5.83	3.24					0.96	1.42	0.04	2.26
GH9	Cellulose															2.16	1.18					1.74	2.07		
GH105	Hemicellulose															0.17				1.08	1.42	0.22	0.25	0.02	0.87
GH106	Hemicellulose																	0.21	0.18	0.05	0.11			0.43	0.90
GH16	Hemicellulose															0.98	0.67			0.01	0.10				
GH2	Hemicellulose					0.02	0.03	0.97	0.76	0.27	0.19			1.71	0.80	1.97	1.22	2.01	1.32	0.33	0.49	0.77	1.11	0.13	1.12
GH26	Hemicellulose															1.67	0.72					1.01	1.43		
GH35	Hemicellulose																			0.54	0.74	1.00	1.54	0.05	0.68
GH36	Hemicellulose	0.67	1.07					0.83	1.14	0.35	0.20							2.04	1.23			2.38	3.12	0.08	2.07
GH78	Hemicellulose																			0.53	0.57	1.41	1.95		
GH97	Hemicellulose																			0.90	1.27	2.18	3.09	0.08	2.47
PL11	Hemicellulose															0.37	0.26			0.22	0.50	0.87	1.11	0.04	1.31
CE12	Pectin																			1.12	1.45	0.53	1.22	0.18	1.77
CE8	Pectin															0.58	0.50					0.83	1.79	0.37	2.55
GH28	Pectin	0.16	0.12																	0.67	0.87	2.16	3.84	0.18	2.76
GH42	Pectin															0.20	0.14								
GH95	Pectin															0.51	0.29			0.08	0.10	1.20	1.62	0.06	1.24
PL1	Pectin															0.14	0.06					0.98	1.96	0.06	1.57
PL10	Pectin																							0.21	0.44
PL9	Pectin															0.05	0.14								
GH13	Starch	0.62	0.39	1.22	0.91	0.63	0.38	2.79	2.36	0.89	0.67	0.77	0.40	1.97	0.88	1.52	0.63	1.54	1.23	0.45	0.58	3.20	4.31	0.31	4.40
GH31	Starch													0.93	0.53	0.28	0.10	1.08	0.82	0.33	0.36	1.73	2.52	0.31	1.46
GH57	Starch																					2.66	3.16	1.13	27.08
GH18	Chitin							1.16	0.96																
GH30	Arabinan															0.29	0.37							1.06	2.44
GH51	Arabinan																			1.27	1.68	3.61	4.43	0.11	1.40
GH53	Arabinan															0.06	0.02			0.24	0.55	2.41	3.33		
GH32	Fructan	2.12	1.14			0.53	0.37	1.48	1.07	2.16	1.79	2.16	1.58	8.88	3.60	6.62	3.27	11.29	8.63	0.80	0.51			0.15	1.84

Scaled fold−change (log10)

1.0

0.5
