# Supplementary material for: Intricate microbiome differences observed in lactating cows across methane intensity phenotypes
Source: ISME Commun. 2026 Jun 7;6(1):ycag155. doi: 10.1093/ismeco/ycag155 (PMC13431278; doi:10.1093/ismeco/ycag155)
Supplement: Supplementary_material_ycag155 [file supplementary_material_ycag155.zip › SF_11.pdf]

	MAG_385		MAG_52		MAG_245		MAG_39		MAG_62		MAG_44		MAG_68		MAG_146		MAG_314		MAG_195		MAG_228		MAG_217			
	LMI	HMI	LMI	HMI	LMI	HMI	LMI	HMI	LMI	HMI	LMI	HMI	LMI	HMI	LMI	HMI	LMI	HMI	LMI	HMI	LMI	HMI	LMI	HMI		
			17.68	13.21			1.65	1.19							0.07	0.03									K00169	Acetate
			18.32	14.66			1.19	0.87							0.06										K00170	
			8.63	6.07			0.71	0.59																	K00171	
			11.67	7.62			1.05	1.11							0.15	0.02									K00172	
											5.04	4.01	6.09	3.24			6.30	4.59							K00625	
															5.18	2.45									K00656	
			9.40	8.04	2.75	1.99	113.16	89.27	5.58	4.71					1.08	0.76					7.22	8.23	0.75	18.02	K00925	
			4.14	2.98			284.79	223.39	1.04	0.92	22.50	14.69	23.48	9.61	3.96	1.97	27.66	19.55			26.24	32.92			K03737	
															0.43	0.35									K00016	Lactate
													127.26	59.12											K03778	
	9.91	7.92			5.17	4.75					7.40	4.81			2.63	1.21									K18332	WLP_additional
															0.43	0.35									K00016	Propionate via acrylate
			0.18	0.09	5.07	3.56																			K01026	
							3.02	2.37																	K00123	WLP
															1.88	1.34									K00125	
															0.20	0.14									K00198	
	1.23	0.97							0.12	0.37	1.07	0.77	2.51	1.07	0.27	0.10	1.41	1.19	0.89	1.01	2.86	3.58	0.07	0.56	K00297	
	0.37	0.39													0.56	0.33					2.66	3.40	0.09	2.39	K01491	
			0.64	0.39					0.78	0.34	0.98	0.37	1.14	0.45											K01500	
	1.09	1.02	2.38	1.65	0.85	0.73	5.74	5.40					1.26	0.65											K01938	
			242.32	193.50					21.89	12.85															K00074	Butyrate
	79.56	59.98					92.94	77.83	14.50	10.12															K00248	
			266.08	207.91			211.57	172.62	42.79	31.92															K00626	
									0.24	0.20															K00929	
			15.58	13.63	34.49	25.61	1.80	1.30	1.32	0.68															K01715	
											30.55	23.27					56.27	46.04							K04072	Ethanol
																							7.30	31.63	K00239	Propionate via succinate
													0.33	0.12									13.84	35.98	K00240	
																							5.64	27.71	K00241	
																			4.69	4.16	9.88	10.80	0.47	10.39	K01676	
			1.50	1.26																					K01678	
	0.64	0.32					2.68	2.09	0.95	0.59	1.06	0.51	0.88	0.38			0.42	0.51							K01679	
																					11.67	15.41			K01847	
			3.96	3.00	3.94	2.74	40.70	31.56	3.34	2.77															K01958	

Scaled fold−change (log10)

2.0
1.5
1.0
0.5
