# Supplementary figures and images for: Intricate microbiome differences observed in lactating cows across methane intensity phenotypes
Source: ISME Commun. 2026 Jun 7;6(1):ycag155. doi: 10.1093/ismeco/ycag155 (PMC13431278; doi:10.1093/ismeco/ycag155)

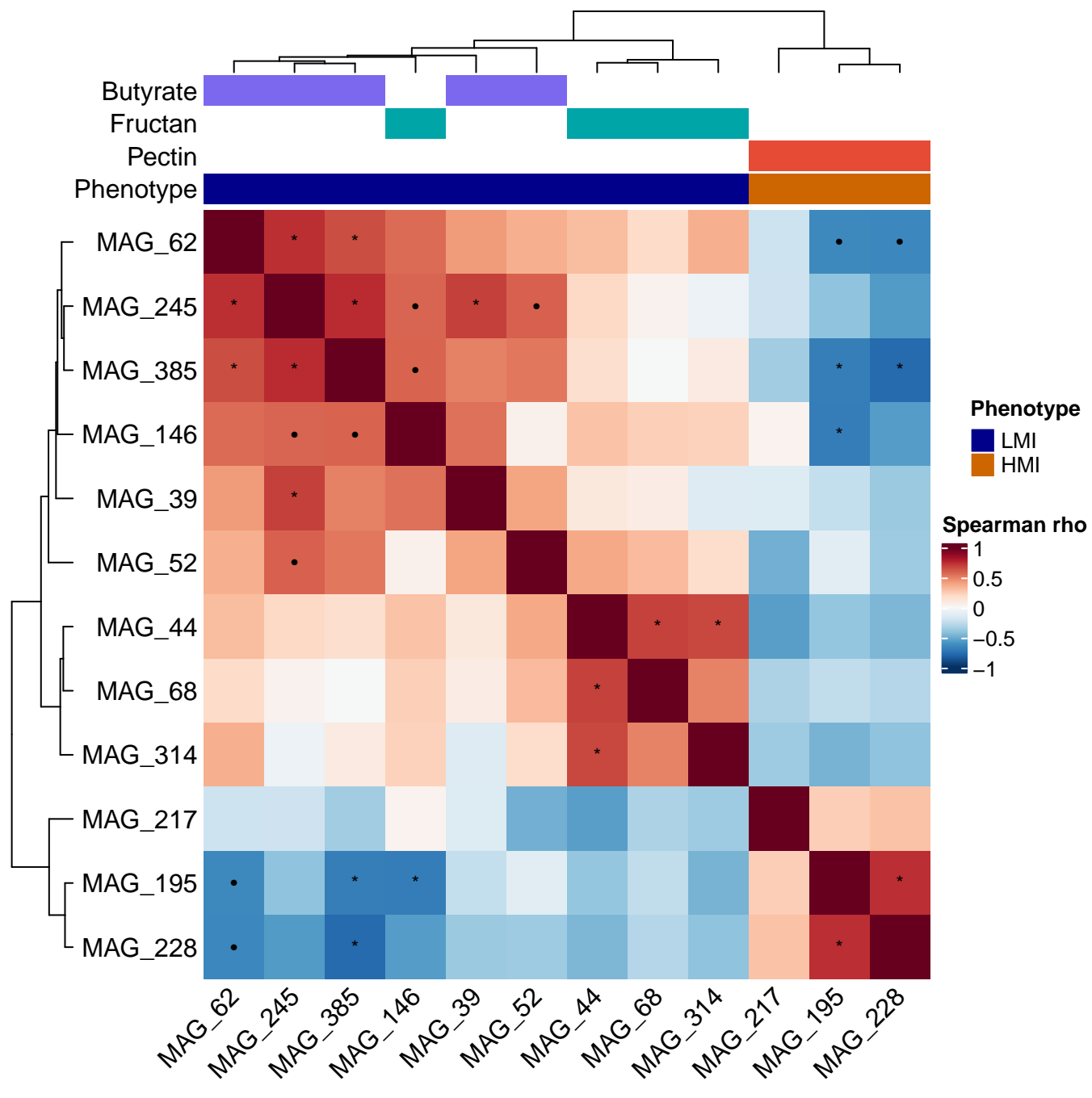

Supplement: Supplementary_material_ycag155 [file supplementary_material_ycag155.zip › SF_13.pdf]

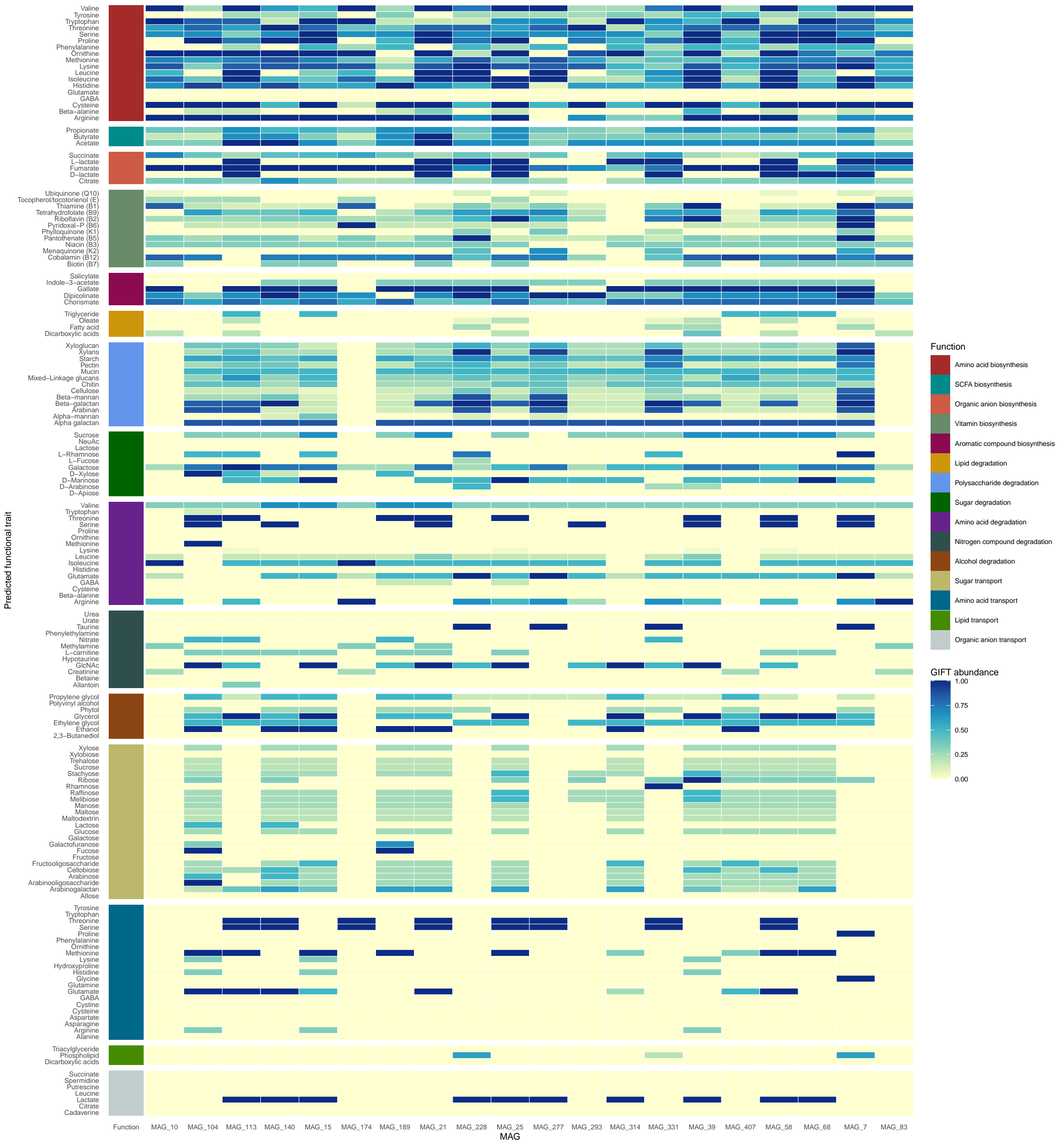

Supplement: Supplementary_material_ycag155 [file supplementary_material_ycag155.zip › SF_14.pdf]

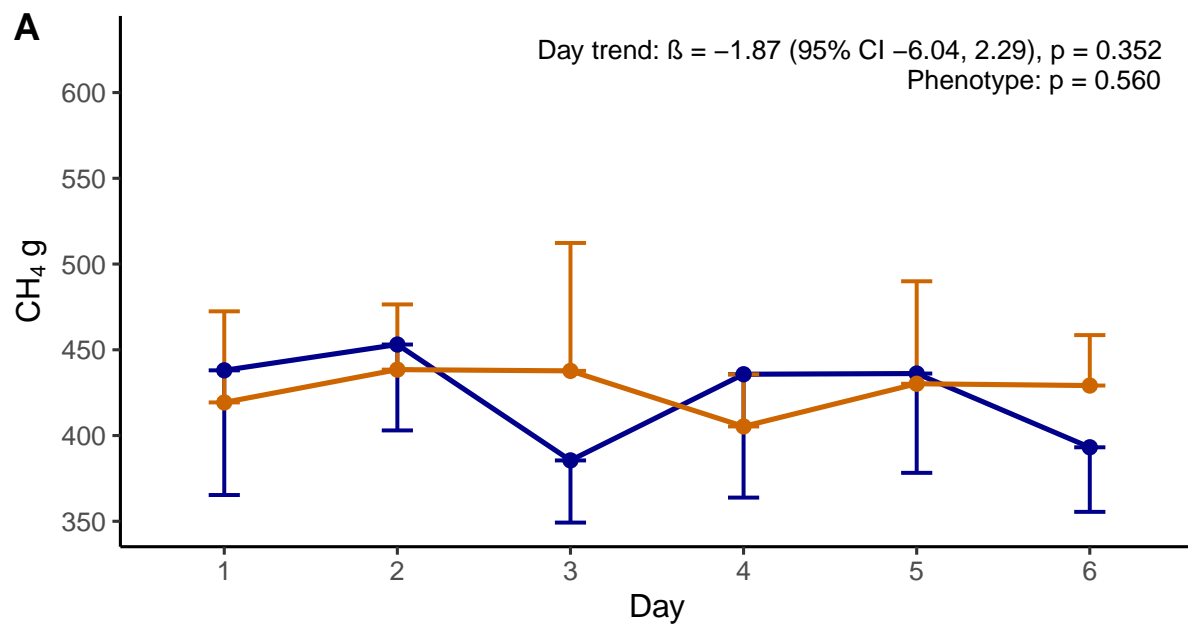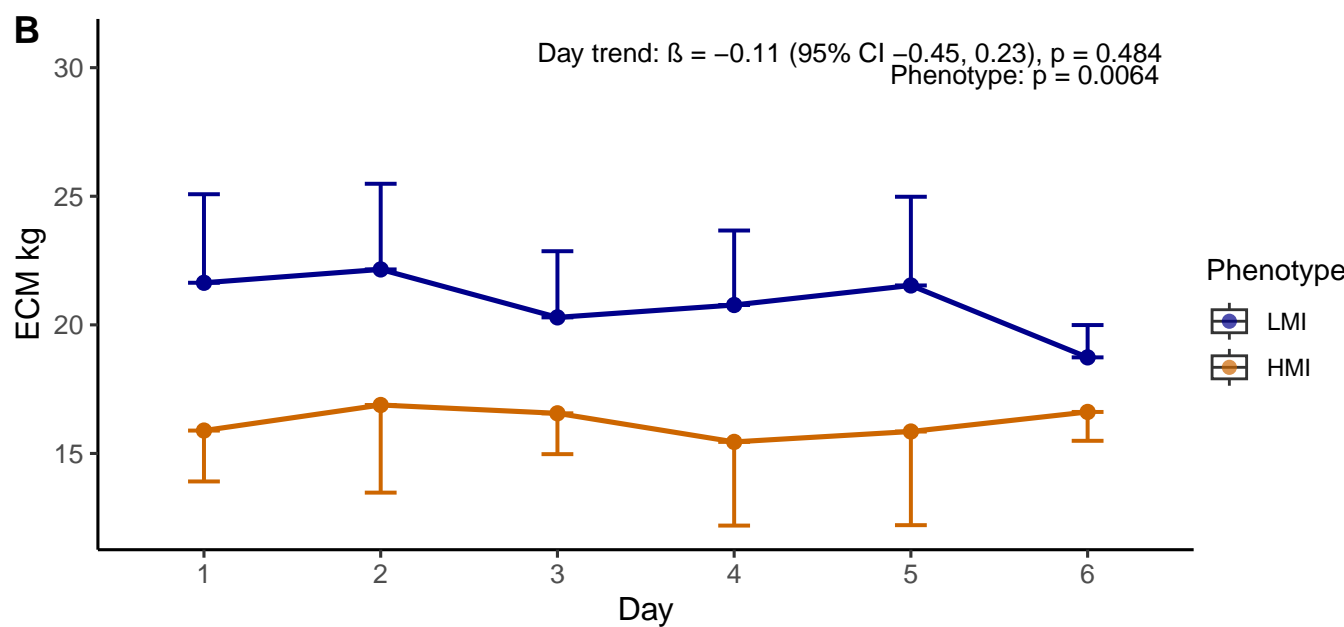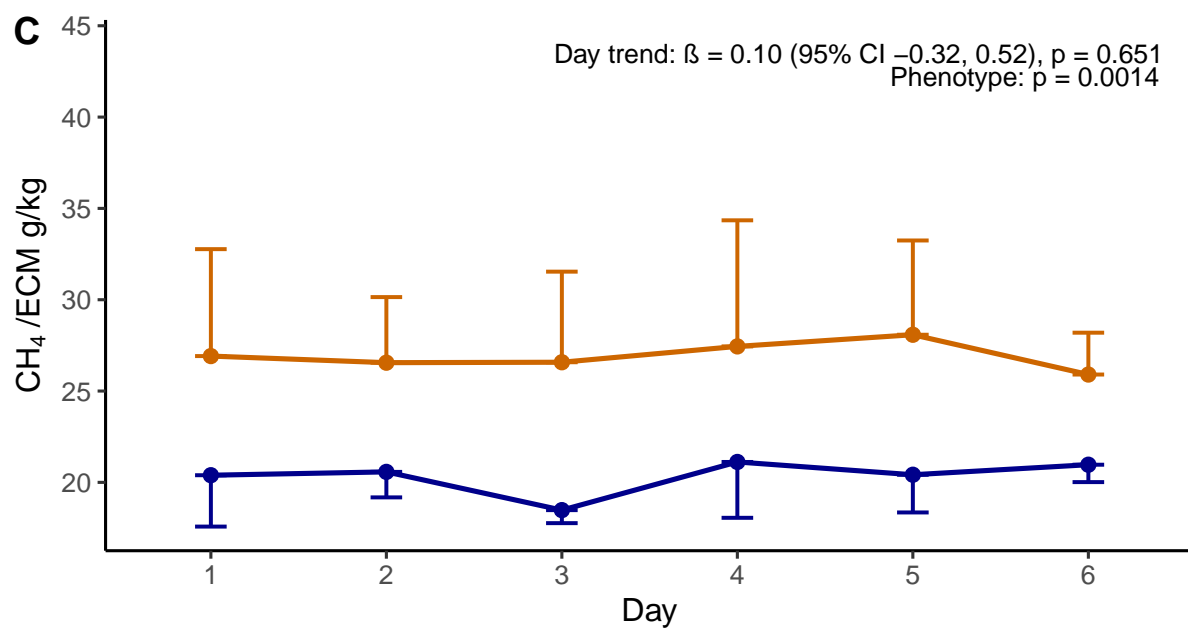

Supplement: Supplementary_material_ycag155 [file supplementary_material_ycag155.zip › SF_1.pdf]

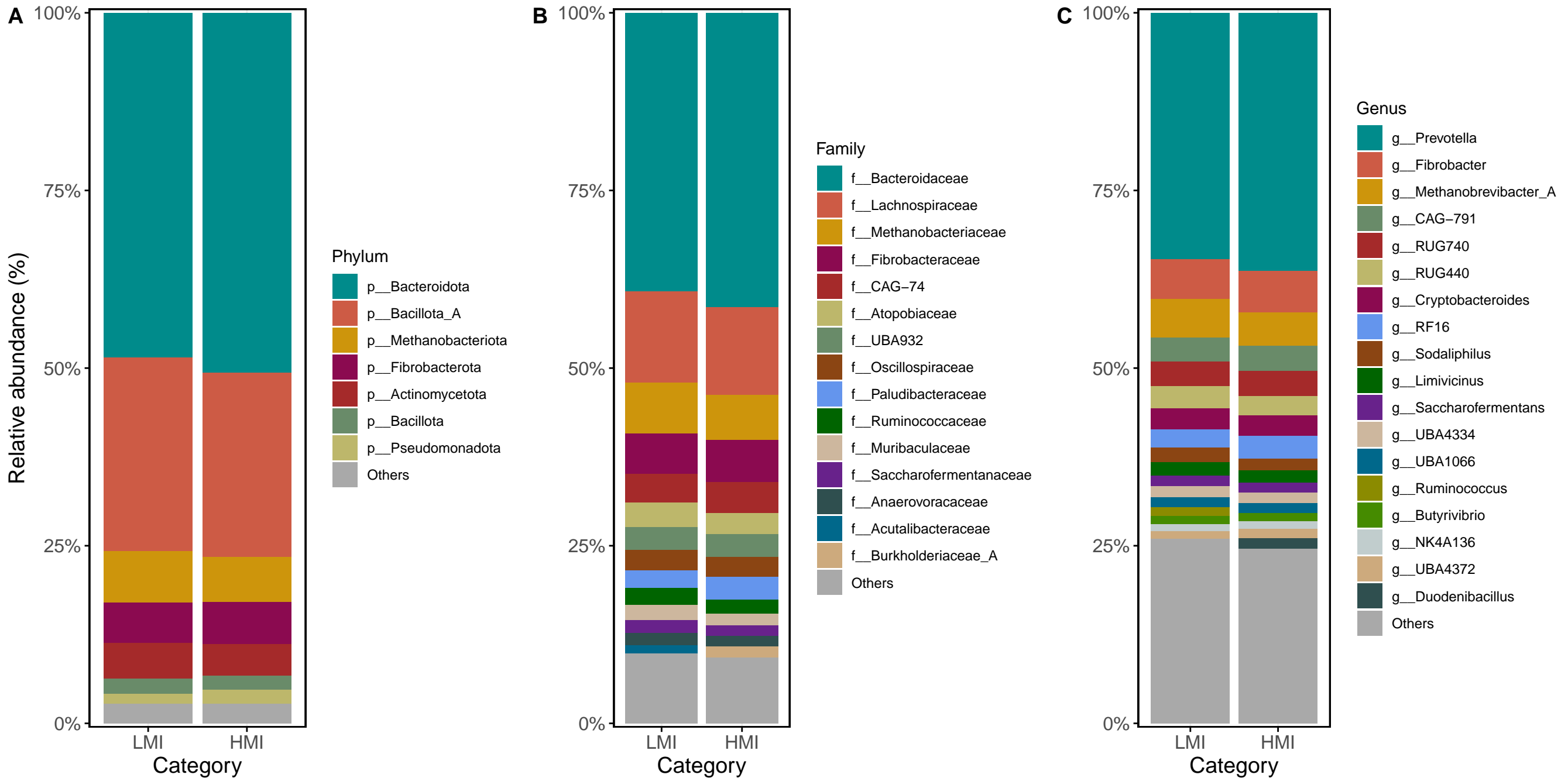

Supplement: Supplementary_material_ycag155 [file supplementary_material_ycag155.zip › SF_2.pdf]

**A**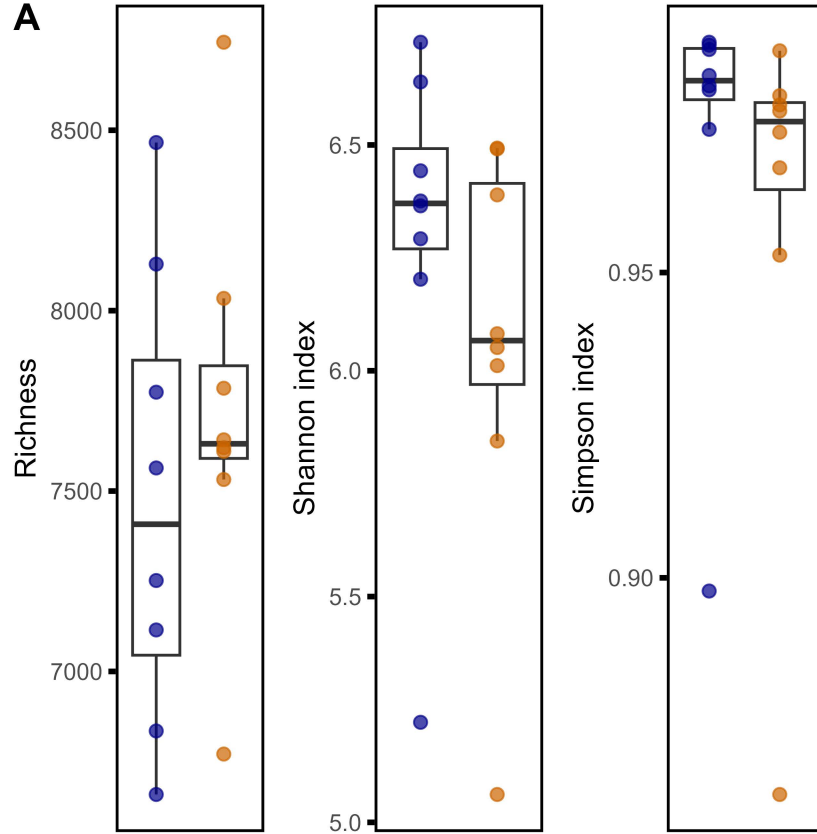**B**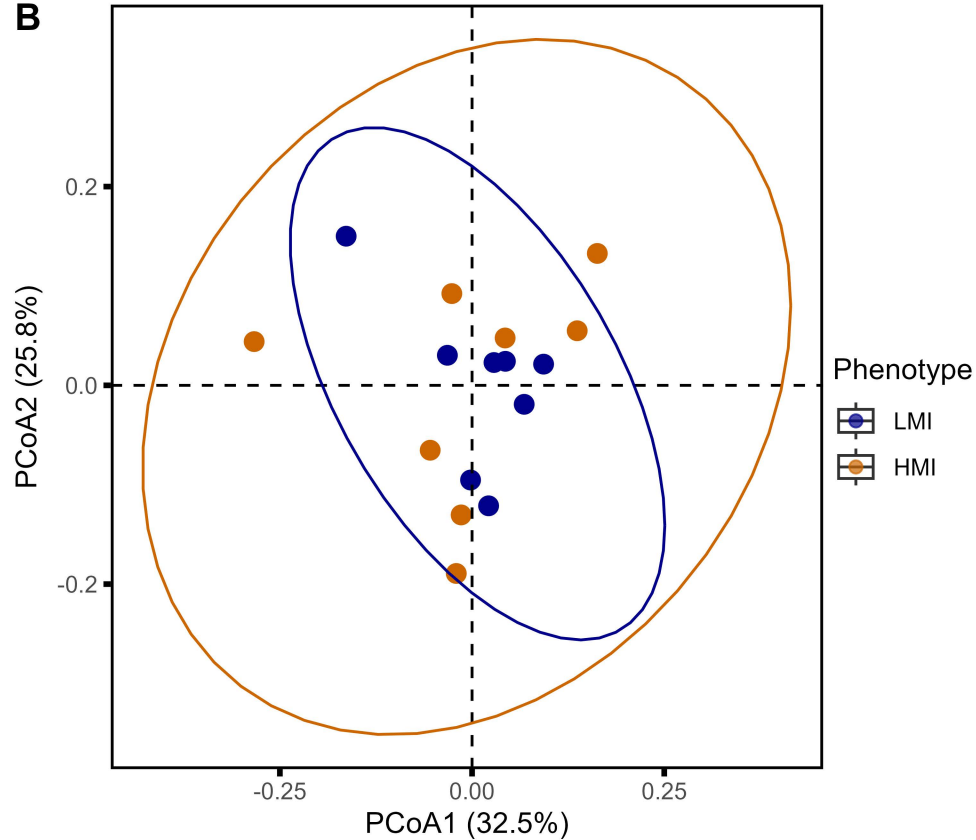

Supplement: Supplementary_material_ycag155 [file supplementary_material_ycag155.zip › SF_3.pdf]

**A**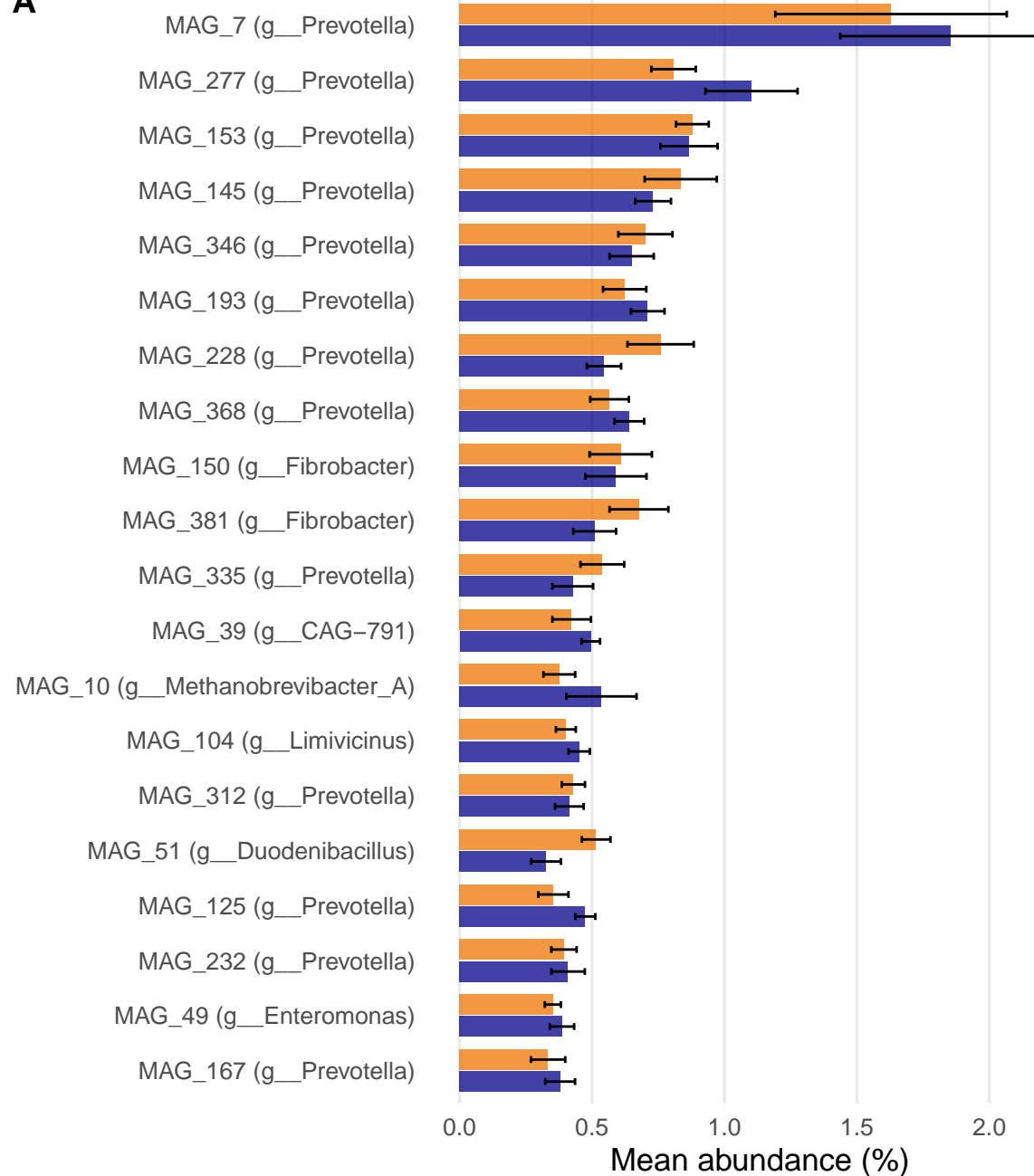**B**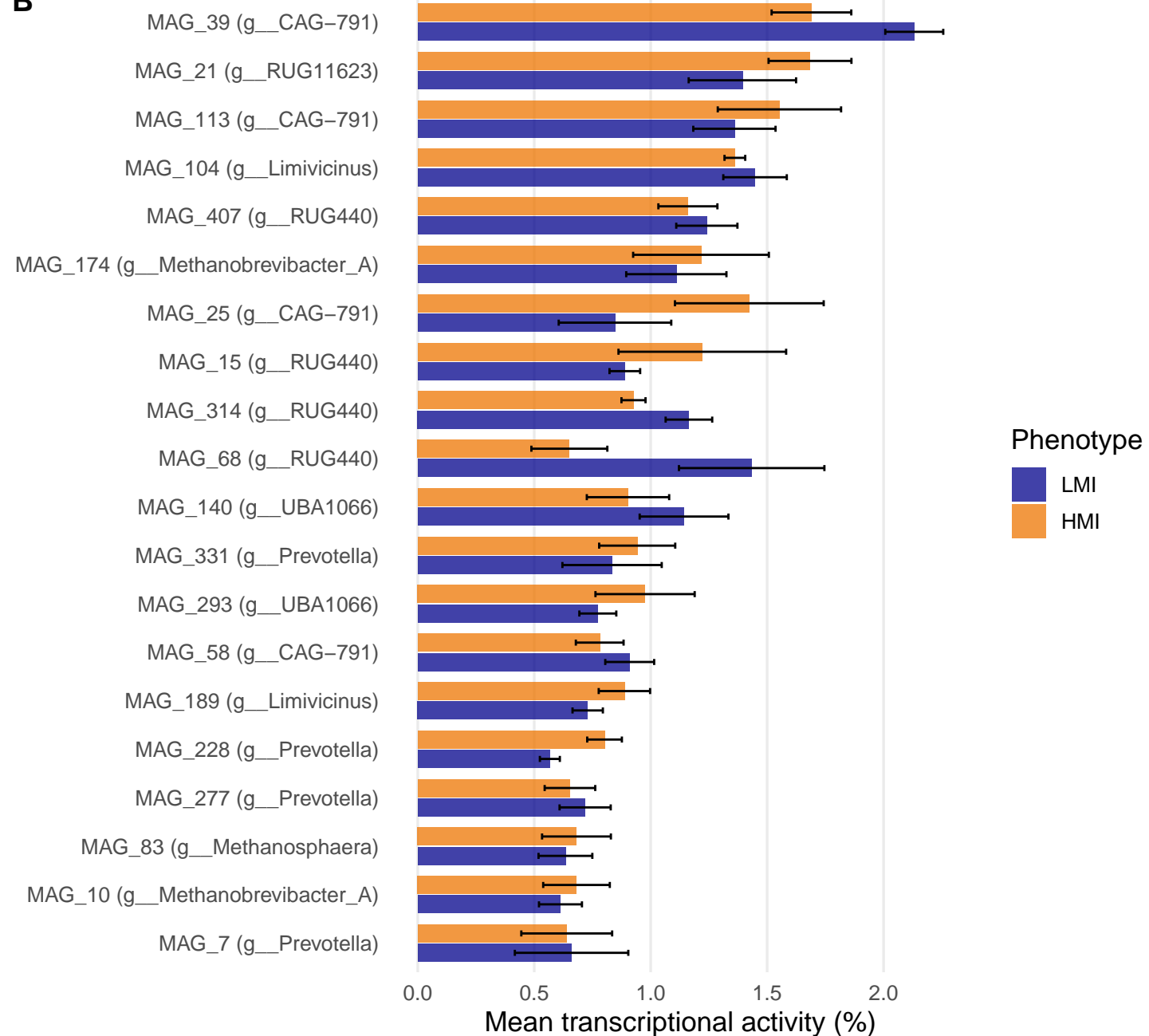

Supplement: Supplementary_material_ycag155 [file supplementary_material_ycag155.zip › SF_4.pdf]

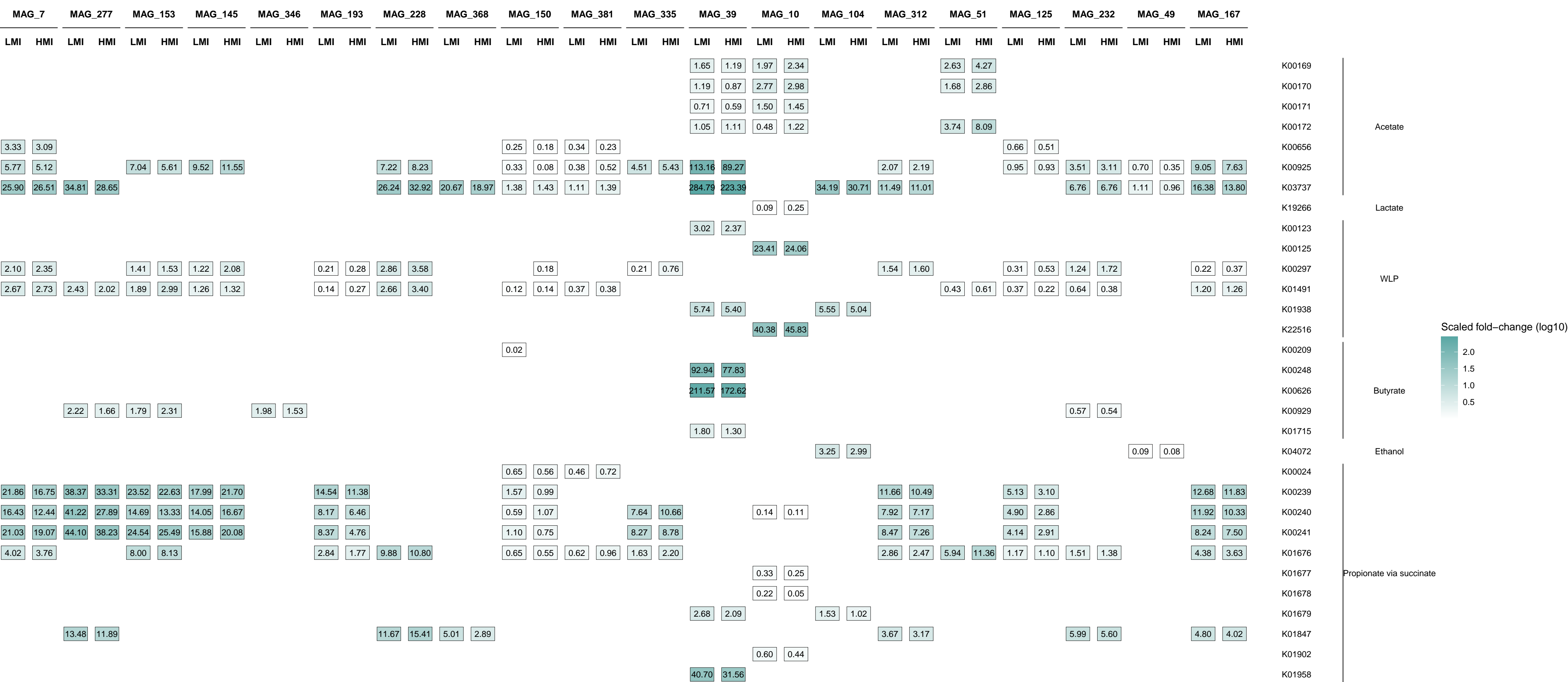

Supplement: Supplementary_material_ycag155 [file supplementary_material_ycag155.zip › SF_7.pdf]
